# Parastomal hernias after cystectomy and ileal conduit urinary diversion: surgical treatment and the use of prophylactic mesh: a systematic review

**DOI:** 10.1186/s12893-022-01509-y

**Published:** 2022-03-29

**Authors:** M. Dewulf, N. D. Hildebrand, S. A. W. Bouwense, N. D. Bouvy, F. Muysoms

**Affiliations:** 1 Department of Surgery, Maastricht UMC+, P. Debyelaan 25, 6229 HX Maastricht, The Netherlands; 2 Department of Surgery, Maria Middelares, Gent, Belgium

**Keywords:** Parastomal hernia, Ileal conduit, Cystectomy

## Abstract

**Background:**

Parastomal hernia after ileal conduit urinary diversion is an underestimated and undertreated clinical entity, which heavily impairs patients’ quality of life due to symptoms of pain, leakage, application or skin problems. As for all gastrointestinal stomata the best surgical repair technique has yet to be determined. Thereby, surgery for ileal conduit parastomal hernias poses some specific perioperative challenges. This review aims to give an overview of current evidence on the surgical treatment of parastomal hernia after cystectomy and ileal conduit urinary diversion, and on the use of prophylactic mesh at index surgery in its prevention.

**Methods:**

A systematic review was performed according to PRISMA-guidelines. The electronic databases Embase, PubMed, Cochrane Library, and Web of Science were searched. Studies were included if they presented postoperative outcomes of patients undergoing surgical treatment of parastomal hernia at the ileal conduit site, irrespective of the technique used. A search was performed to identify additional studies on prophylactic mesh in the prevention of ileal conduit parastomal hernia, that were not identified by the initial search.

**Results:**

Eight retrospective case-series were included for analysis, reporting different surgical techniques. If reported, highest complication rate was 45%. Recurrence rates varied highly, ranging from 0 to 80%. Notably, lower recurrence rates were reported in studies with shorter follow-up. Overall, available data suggest significant morbidity after the surgical treatment of ileal conduit parastomal hernias. Data from five conference abstracts on the matter were retrieved, and systematically reported. Regarding prophylactic mesh in the prevention of ileal conduit parastomal hernia, 5 communications were identified. All of them used keyhole mesh in a retromuscular position, and reported on favorable results in the mesh group without an increase in mesh-related complications.

**Conclusion:**

Data on the surgical treatment of ileal conduit parastomal hernias and the use of prophylactic mesh in its prevention is scarce. Given the specific perioperative challenges and the paucity of reported results, more high-quality evidence is needed to determine the optimal treatment of this specific surgical problem. Initial results on the use of prophylactic mesh in the prevention of ileal conduit parastomal hernias seem promising.

**Supplementary Information:**

The online version contains supplementary material available at 10.1186/s12893-022-01509-y.

## Background

Radical cystectomy with ileal conduit urinary diversion remains the cornerstone of curative treatment of patients with urothelial carcinoma of the bladder [1]. One possible long-term postoperative complications after this type of surgery is parastomal hernia at the ileal conduit site. Parastomal hernia is defined as the protrusion of contents of the abdominal cavity through the abdominal wall, in the direct proximity of a colostomy, ileostomy or ileal conduit stoma [2]. Overall, the incidence of parastomal hernia at any type of stoma site after 12 months is estimated to be around 30% [3]. For ileal conduit urinary diversion a systematic review reported an estimated incidence of parastomal hernia of 17% [4].

To date, numerous surgical techniques to treat parastomal hernia have been described, such as local suture repair, relocation of the stoma or mesh repairs (with onlay, retromuscular, retroperitoneal or intraperitoneal mesh position). Regarding mesh repairs, various configurations have been proposed (Keyhole, Sugarbaker or pre-shaped meshes) [5, 6]. Recurrence rates after parastomal hernia repair of colo- and ileostomy remain high with up to 69% after 1 year [5–8]. Evidence remains limited and is mostly based on small retrospective case series. For end colostomy, prophylactic mesh placement has proven to be an effective technique in the prevention of parastomal hernia, and has gained acceptance in recent years [3].

Regarding parastomal hernia after ileal conduit urinary diversion, evidence is lacking. This type of repair poses specific perioperative challenges including stripped peritoneum below the arcuate line, short mesentery of the conduit complicating lateralization of the stoma, difficult perioperative identification of the ileal conduit loop due to longstanding collapse, concomitant midline incisional hernias and presence of ureteric anastomoses. Furthermore, evidence on prophylactic mesh in this type of surgery is limited. This stresses the need to further evaluate and optimize the surgical treatment of this specific type of parastomal hernia.

## Objectives

Until now, systematic reviews on the surgical treatment of parastomal hernias have evaluated available evidence on all types of stomas. However, as mentioned, repair of ileal conduit parastomal hernias pose specific perioperative challenges. The aim of this systematic review is to collect all current evidence on the surgical treatment of parastomal hernia after ileal conduit urinary diversion. Furthermore, available literature on the use of prophylactic mesh in the prevention of ileal conduit parastomal hernias is collected.

## Methods

### Search strategy

This systematic review was written according to the Preferred Reporting Items for Systematic Reviews and Meta-analysis (PRISMA) guidelines [9], and was registered in the International Prospective Register of Systematic Reviews (PROSPERO) database on December 16, 2020. Considering the manuscript being a literature review, there was no obligation to seek approval by the Institutional Review Board. The electronic databases MEDLINE (through PubMed), Embase, Web of Science, and Cochrane Central Register of Controlled Trials (CENTRAL) were searched for eligible articles. ClinicalTrials.gov and the International Clinical Trials Registry Platform (ICTRP) portals were searched to identify ongoing research on the matter. Combining predefined search terms and operators ‘OR’ and ‘AND’ resulted in the following search: *(((((cystectomy) OR urinary diversion) OR ileal conduit) OR urostomy)) AND ((hernia) OR parastomal hernia).* Search filters were not applied. The search was conducted in January 2021 and repeated in November 2021. Reference lists of reviews on the topic and included full text articles were manually screened by two reviewers (NH, MD) to identify additional sources. Abstract books of annual meetings of the European Hernia Society, European Association of Endoscopic Surgery and American Hernia Society were screened for conference abstracts.

After exclusion of duplicates, results were screened by title, abstract and subsequently evaluation of full text. When no full text was available, authors were contacted to provide additional information. When only a subset of patients was eligible for inclusion, authors were contacted to provide specific data on the subgroup. The predefined study protocol can be found in Additional file 1: Appendix S1, a detailed description of the literature search is added as Additional file 2: Appendix S2. To identify additional sources on the use of prophylactic mesh in the prevention of parastomal hernia that were not identified by the initial search, a new search was performed in November 2021 by adding the terms *‘prophylactic mesh’* and *‘prevention’* to our initial search using the operator ‘OR’.

### Study selection

Two reviewers (NH, MD) independently screened studies according to the predefined inclusion and exclusion criteria. Studies were considered eligible if they included patients underwent surgical treatment of parastomal hernia at the ileal conduit site. No exclusions were made based on study design, type of surgical treatment, or language. Exclusion criteria were patients younger than 18, other types of urinary diversion besides ileal conduit stoma, animal studies, and case series reporting on less than 5 patients. Studies on the use of prophylactic mesh were collected separately.

### Outcome measurements

Primary outcome was incidence of postoperative complications according to Clavien-Dindo-classification [10] within 30 days of surgery. Secondary endpoints were recurrence rates after 1 year, length of hospital stay, and 30-day reoperation and readmission rates. For the studies on prophylactic mesh parastomal hernia rate was defined as the primary endpoint, incidence of mesh-related complications as the secondary endpoint.

### Data extraction

A data extraction sheet was developed using Microsoft Excel (Microsoft, Washington, USA). Data retrieved were baseline characteristics (study period, sample size, age, sex), surgical details (approach, technique, mesh position, type of mesh), perioperative data (operation time, estimated blood loss), and primary and secondary endpoints of postoperative outcomes. Given the variety of surgery techniques, a pooled analysis of results was not performed.

### Assessment of methodological quality

Our study protocol proposed the use of the Risk of Bias in Non-randomized Studies—of Interventions (ROBINS-I)-tool [11] to assess methodological quality of included studies. However, given that none of the included studies were comparative, methodological quality was evaluated using the methodological index for non-randomized studies (MINORS)-tool [12]. Both reviewers (NH, MD) independently assessed the studies. Disagreement was dissolved through discussion, consultation of the senior authors was performed if necessary.

## Results

**Fig. 1:**
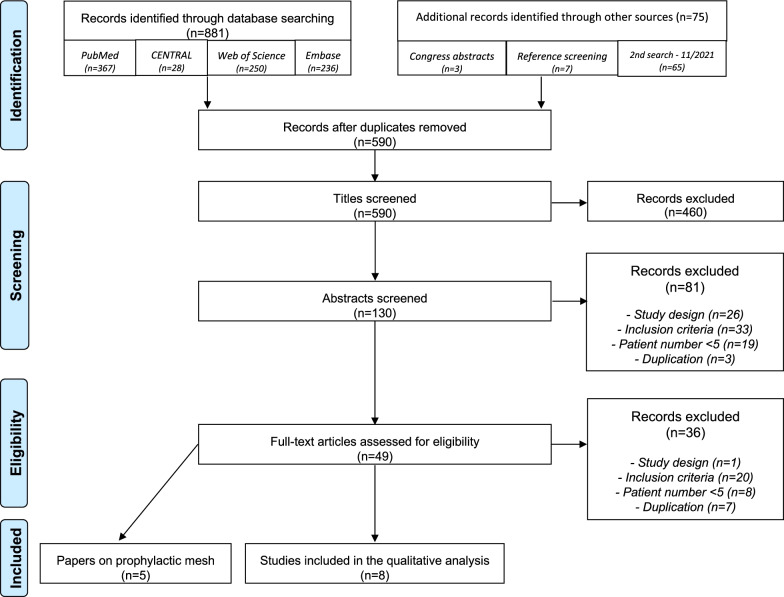
PRISMA flow diagram of study selection

Search details and study selection are illustrated in a PRISMA flow diagram (Fig. 1). Authors were contacted to provide additional data on 15 conference abstracts [13–27] and 9 full-text articles with subgroups of ileal conduit patients [28–36]. However, no additional data was available or provided. Eventually, 8 full-text articles met the inclusion criteria and were included in the qualitative analysis [37–44]. An overview of study characteristics, surgical details and postoperative outcomes is shown in Table 1. Details on five conference abstracts that met the inclusion criteria are depicted in Table 2 [23–27]. Studies reporting on the use of prophylactic mesh in the prevention of ileal conduit parastomal hernia are summarized in Table 3.

**Table 1:** Study characteristics, surgical details and postoperative outcomes of included full-text articles

Study characteristics*						Surgical details					Postoperative outcomes*						
Author & Year	Country	Study period	Sample size	Mean age	Male (%)	Surgical technique	Appr	Mesh repair	Mesh position	Mesh type	Post-OP compl. rate	CD Grade > II	Rec. rate	Length of follow-up	Type of follow-up	Length of stay	30-day re-operation rate
Franks 2001	USA	NR	6	(65–83)	NR	Keyhole	Open	Yes	Onlay	small pore, heavy weight	NR	NR	0%	26 m (2–42)	Clinical + CT	2.5 d (2–5)	0%
Helal 1997	USA	1990–1996	19	64.3(41–79)	26%	Re- location	Open	Yes (^†^)	Onlay	small pore, heavy weight	NR	NR	11%	23.4 ± 15.25 m	NR	NR	NR
Ho 2004	UK	1982–2001	15	(34–82)	47%	Onlay mesh repair (^‡^)	Open	Yes	Onlay	small pore, heavy weight	13%	13%	7%	15 m (1–72)	NR	4 d (2–14)	13%
Lopez-Cano 2021	Spain	2012–2018	20	71 (SD:9.07)	85,0%	Onlay mesh repair	Open (16) Lap. (4)	Yes	Onlay	synthetic, non- absorbable	45.0%	10.0%	NR	6 m	NR	NR	NR
Mäkäräinen- Uhlbäck** 2021	Finland	2007–2017	18 (KH)	70 ± 9	44,4%	Keyhole	Open (7) Lap. (11)	Yes	NR	NR	33.3%	11.1%	22.2%	49 m ± 34	NR	17.8 ± 50.1 days	5.6%
			10 (SB)	77 ± 6	60,0%	Sugar- baker	Open (2) Lap. (8)	Yes	NR	NR	10.0%	0.0%	10.0%	27 m ± 21	NR	6.3 ± 3.7 days	0.0%
Rodriguez- Faba 2011	Spain	2000–2006	19	63 (49–79)	84%	Re- location	Open	Yes (^§^)	Intra- peritoneal	large pore, light weight	26%	5%	21%	55 m	NR	7 d(1–25)	5%
Safadi 2004	USA	1998–2001	5	66 (54–77)	100%	Keyhole	Lap	Yes	Intra- peritoneal	ePTFE	0%	0%	80%	11.6 m (6–10)	Clinical	4.4 d (2–6)	0%
Tully 2019	Germany	2009–2015	40	NR	63%	3D funnel shape	Open	Yes	Intra- peritoneal	large pore, light weight	3%	3%	7%	29 m (IQR 16–63)	Clinical + US	NR	0%

*Numbers within brackets indicate ranges, unless otherwise stated

^†^Mesh was used at the previous stoma site for 2 patients with large defects

^‡^A lateral approach was used, where only the lateral part of the parastomal hernia was covered with mesh

^§^Mesh was used at previous stoma site

**National cohort

*NR* not reported,* lap.* laparoscopic,* compl.* complication,* rec.* recurrence,* m* months,* IQR* interquartile range,* CT *computed-tomography scan,* US* ultrasonography,* CD Clavien-Dindo*

**Table 2:** Study characteristics, surgical details and postoperative outcomes of included conference abstracts

Study characteristics*						Surgical details				Postoperative outcomes*				
Author	Country	Study period	Sample size	Mean age	Male (%)	Surgical technique	Approach	Mesh repair	Mesh position	Post- operative compl. rate	Rec. rate	Length of follow-up	Type of follow-up	Length of stay
Antor 2017	France	2006–2015	9	63 (59–83)	NR	NR	Lap	Yes	Intra-peritoneal	0%	22%	27 m (7–106)	Clinical + PC	6d (4–13)
Davis 2012	Canada	2005–2010	11	63.9 (47–79)	36%	NR	Lap	Yes	NR	NR	27%	19.1 m (1–62)	NR	6.3d (1–12)
Jaipuria 2020	India	2018–2019	6	67	67%	MS	RA	Yes	Intra-peritoneal	NR	0%	10 m	NR	2d
Shakir 2020	USA	2017–2019	7	71	29%	Keyhole	RA	Yes	Intra-peritoneal	29%	0%	90 d	NR	4d
Von Bodman 2012	Germany	2009–2011	13	70	54%	3D funnel shape	Open	Yes	Intra-peritoneal	31%	8%	23 m	Clinical + US	NR

*Numbers within brackets indicate ranges, unless otherwise stated

*NR* not reported,* MS* modified Sugarbaker,* lap.* laparoscopic,* RA* robotic-assisted,* compl.* complications,* rec. *recurrence,* m* months,* PC* phone call,* US ultrasonography*

**Table 3:** Study characteristics, surgical details and postoperative outcomes of literature on prophylactic mesh placement in primary radical cystectomy and ileal conduit urinary diversion

General characteristics*								Surgical details*				Postoperative outcome*				
Author & Year	Country	Study period	Study design	Sample size	Mean age	Male (%)	MIN- ORS	Tech- nique	Appr	Mesh position	Mesh type	Length of follow-up	Type of follow-up	PSH rate	Compl. rate	Mesh- related compl
Donahue 2016	USA	2013–2015	RS	33	NR	51.5%	**5**	Key-hole	Open	Retro-rectus	large pore, light weight	297 days	Clinical + CT	Clin: 3% CT: 18.2%	NR	0.0%
Liedberg 2020	Sweden	2012–2017	RCT	C:124 M:118	C: 71 M: 71	C: 79% M: 77%	**21**	Key-hole	Open	Retro-rectus	large pore, light weight	3 years	Clinical + CT	C: 29.3% M: 10.2%	C: 41.5%M: 43.1%	NR
Styrke 2015	Sweden	2003–2012	RS	58	69 ± 7	59.0%	**10**	Key-hole	Open	Retro-rectus	large pore, light weight	32mon	Clinical + CT	14.0%	NR	0.0%
Tenzel 2018	USA	2010–2017	RS	C: 20 M: 18	68	74.0%	**10**	Key-hole	Rob	Retro-rectus	synthetic resorbable/bio	C: 21mon M: 11mon	CT	C: 5% M: 0%	NR	0.0%
*Jian 2021 (CA)*	*USA*	*2019–2021*	*RS*	*38*	*NR*	*NR*	***NA***	*Key-hole*	*Rob*	*Retro-rectus*	*medium weight, mono-filament*	*5 months (n* = *21)*	*CT (n* = *21)*	*5.0%*	*NR*	*0.0%*

*: numbers within brackets indicate ranges, unless otherwise stated

*CA* conference abstract, *C* control (no mesh), *M* mesh, *RS* retrospective *PS* prospective, *NC* nationwide cohort, *NR* not reported, *m* months, CT Computed-Tomography scan, *SD* standard deviation, *US* ultrasonography

### Surgical treatment of ileal conduit parstomal hernias

All studies were retrospective. Most data comes from European [25, 26, 37–39, 45] and North-American centers [23, 27, 40–42]. One conference abstract is from India [24]. A total of 124 patients are included in full-text articles and 46 patients in conference abstracts, with sample sizes ranging from 5 to 40.

Techniques described in the full-text papers were onlay mesh repair covering only the lateral part of the stoma (n = 15) [39], onlay mesh repair with a non-specified technique (n = 20) [44], relocation (n = 38) [37, 41], keyhole technique (n = 29) [40, 42, 43], Sugarbaker repair (n = 10) [43] and use of a 3D-funnel-shapedmesh (n = 40) [38]. Additionally, in conference abstracts the keyhole-technique (n = 7) [27], and 3D-funnel-shaped mesh (n = 13) [26] were presented. Two conference abstracts described their technique as ‘intraperitoneal mesh’ and ‘laparoscopic repair’, but did not further specify their technique [23, 25]. Approaches used were predominantly open (n = 137) [26, 37–41, 43, 44], laparoscopic (n = 49) [23, 25, 42–44], and robot-assisted (n = 13) [24, 27]. Mesh placement was performed in all studies, the mesh position was either onlay (n = 60) [39–41, 44] or intraperitoneally (n = 99) [24–27, 37–39, 42]. Two groups did not specify the mesh position (n = 39) [23, 43]. Only synthetic, non-absorbable meshes were used. Three groups used small pore, heavy weight mesh (n = 40) [39–41], one used ePTFE (n = 5) [42], and two reported the use of large pore, light weight mesh (n = 59) [37, 38].

Recurrence rates ranged from 0 to 80% in included full-text articles [37–44], and from 0 to 22% within conference abstracts [23–27]. Length of follow-up varied from 12 to 55 months within full-text articles [37–44], and from 90 days to 27 months for conference abstracts [23–27]. Follow-up, if reported, consisted of clinical examination, CT-scan or ultrasound [25, 26, 38, 40, 42]. Overall postoperative complications ranged from 0 to 45% [25–27, 37–39, 42–44]. Major complications (defined as Clavien-Dindo > II) occurred in 0–11% of the cases [25, 27, 37–39, 42–44]. Length of stay in the hospital ranged from 2 to 7 days, with a maximum upper limit of 25 days [23–25, 27, 37, 39, 40, 42–44]. 30-day reoperation rate was, if reported, 0–11% [26, 37–40, 42, 43]. 30-day readmission rate was only reported within one study, where no readmissions occurred [38].

### Prophylactic mesh in the prevention of ileal conduit parastomal hernias

Available literature on the use of prophylactic mesh consists of 1 randomized controlled trial, 3 retrospective cohort studies, and 1 conference abstract. Our initial search identified the four published studies, additional search identified one conference abstract on the topic. All of them report on results of a retrorectus keyhole mesh. Three studies use it in open surgery, 2 of them report on robotic-assisted surgery. In none of the reported data mesh-related complications were seen during a follow-up period between 5 months and 3 years, and favorable results regarding incidence of parastomal hernia in the mesh group are noted. In the Swedish randomized controlled trial, published by Liedberg et al. in 2020, a significant decrease in parastomal hernia rates was seen during the follow-up period of 3 years, without an increase in complications. A significant increase in operative times was noted in the patient group that was treated with prophylactic mesh at index surgery.

### Quality assessment

Results of the quality assessment of included full-text articles using the MINORS-tool [12] are shown in Table 4. Overall, the quality of evidence was poor, mainly due to the lack of prospective design, absence of study size calculation, and non-blinded assessment of results.

**Table 4:** Summary of MINOR-score for all included full-text articles

MINORS	Items*								
Article	A clearly stated aim	Inclusion of consecutive patients	Prospective collection of data	Endpoints appropriate to the aim	Unbiased assessment of the study endpoint	Appropriate follow-up period	Loss to follow-up < 5%	Prospective calculation of study size	Total
Franks 2001	2	0	0	1	0	2	0	0	5
Helal 1997	2	0	0	1	0	2	0	0	5
Ho 2004	2	1	1	1	0	2	0	0	7
Lopez-Cana 2021	2	1	2	2	0	2	1	0	10
Mäkäräinen- Uhlbäck 2021	2	0	0	2	0	2	1	0	7
Rodriguez-Faba 2011	2	2	1	1	0	2	0	0	8
Safadi 2004	2	1	1	1	0	2	2	0	9
Tully 2019	2	2	1	2	0	2	1	0	10

*For each item a score of 0 (not reported), 1 (reported but inadequate), or 2 (reported and adequate) can be given. The global ideal score for non-comparative studies is defined as being 16

*MINORS *methodological index for non-randomized studies

## Discussion

Numerous techniques have been proposed in the surgical treatment for parastomal hernias [3, 5, 6]. These can be grouped into local suture repairs, relocation of the stoma or mesh-based repairs. For mesh placement different anatomical positions can be used, being onlay, retromuscular, or intraperitoneal. Thereby, various configurations of the mesh in relation to the stoma have been presented, such as keyhole (stoma going through the mesh), Sugarbaker (lateralizing the stomal loop using an intraperitoneal mesh), or retromuscular Sugarbaker (lateralizing the stomal loop in the retromuscular plane) [3]. Surgical approach can be grouped into open, laparoscopic or robotic-assisted. Despite this variety of surgical techniques recurrence rates after parastomal hernia repair for colo- and ileostomy patients remain high, with rates of up to 69% [5–8].

## Main results

Two studies reported their results on relocation for ileal conduit parastomal hernia treatment [37, 41]. Intraperitoneal mesh was used at the previous stoma site either in all patients [37], or patients with a large defect [41]. Remarkably, Helal, who only used mesh in two out of 19 patients, reported lower recurrence rates at the old stoma site, when compared to mesh repair (11% vs. 21%). However, follow-up was significantly longer in the paper by Rodriguez-Faba, which can attribute to this difference in recurrence rates. In case of ileal conduit urinary diversion, relocation poses some specific challenges due to ureteric anastomoses and short meso of the ileal conduit, when compared to colo- or ileostomies.

One study reported on the use of onlay mesh via lateral incision and subcutaneous dissection for ileal conduit patients [39]. In this technique, only the lateral part of the stoma was covered with mesh. Complication rate seemed acceptable with 11%, though all complications were major. Reported recurrence rates were lower compared to reported numbers in patients with colo- or ileostomy (6.7% vs 15.2–25.9%) [6, 30]. Given the—in comparison—short follow-up period (15 months), small sample size, and partial coverage of the hernia, these findings must be taken with caution.

Regarding local mesh-based repairs, both keyhole (either flat mesh or 3D funnel-shaped mesh) and modified Sugarbaker techniques have been proposed. For keyhole repair we found open, laparoscopic and robotic-assisted approaches for our patient group of interest. Keyhole repair in general was presented within the nationwide cohort study by Mäkäräinen-Uhlbäck [43]. Overall complications were 33.3%, one third of which were major (n = 18). Recurrence rates were slightly lower (22.2%) to that presented by the same group for their national cohort for colo- and ileostomy patients (36.0% vs. 33.0%) [7]. Laparoscopic keyhole repair, by Safadi et al. showed no postoperative complications for ileal conduit patients, while this was 75.0% for gastrointestinal stomata operated on by the same group [42]. On the contrary, recurrence rates were 80% within 6 months after surgery, and 25% in colo- and ileostomy patients. This recurrence rate for ileal conduit patients is significantly higher than published data in a meta-analysis on laparoscopic repair of all types of stoma (27.9%) [5]. The two groups presenting a ‘laparoscopic approach’ without further clarification for mesh placement, had recurrence rates of 22.2–27.3% [13, 17].

Open keyhole repair as reported by Franks resulted in a recurrence rate of 0.0% after median follow-up of 26 months [40]. Complications are not described. Open keyhole repair of other types of parastomal hernias also had relatively low recurrence rates of 7.2% within a meta-analysis [6]. The use of funnel-shaped meshes, which can be considered as a specific type of keyhole repair, was presented by two German groups [26, 38]. Tully actually had the biggest sample size of patient focusing on ileal conduit, consisting of 40 patients. Complications ranged from 2.5 to 30.8%. Existing literature on this type of mesh repair for all stoma types showed complications ranging from 8.3 to 20.6% [31, 46, 47], so their findings can be considered proportionate. Recurrence rates were roughly in line with evidence for a mixed patient group (7.5–7.7% vs. 9.3–12.5%) [22, 26, 31, 38].

Evidence on the use of the modified Sugarbaker repair is limited. A small patient series showed recurrence rates of 0.0% without any complications in 6 patients, though follow-up was only 10 months [27]. Another small group of patients within a nationwide cohort that was treated with Sugarbaker repair also suggested favorable results and low recurrence rates of 10.0% [43] Preferable outcomes of Sugarbaker over Keyhole repair have been described elsewhere [3, 5–8]. We found one other national cohort that also included a subgroup of ileal conduit parastomal hernia patients [44]. Even though the study period was 6 years only 20 hernia repairs for ileal conduit patients were performed nationally. Recurrence was not reported for urostomy patients, but complications were relatively high with 45.0%. Overall, data on this type of repair in ileal conduit patients is too limited to retain this conclusion in this specific patient group.

## Limitations

This review is subject to several limitations. Firstly, the reported incidence of parastomal hernias in general [8], and after ileal conduit urinary diversion specifically is low [3, 4]. Even nationwide cohort studies on parastomal hernia repair do not exceed 235 patients within 10 years [7, 8]. This, in combination with a broad variety of techniques, compromises sample sizes for study groups. Likewise, in addition to the studies presented, we found 17 case reports and 16 small (n < 5) case series on all types of stoma patients, where novel or partly modified techniques were presented. The results thereof were beyond the scope of this review. Secondly, the broad variety of techniques also made pooling of results impossible. None of the included studies mentioned size of the hernia, which might also influence complication and recurrence rates. This limits our possibilities to draw firm conclusions on the matter. Furthermore, the poor methodological quality of included full-text articles poses another limitation to this review. All included articles had a retrospective design and low MINORS-scores.

The surgical treatment of parastomal hernias after cystectomy and ileal conduit urinary diversion offers some specific challenges.

Firstly, in this condition often a concomitant midline incisional hernia is present. This may highly influence the technique of choice to repair the parastomal hernia. If repair of the midline incisional hernia requires component separation techniques [48], we have a habit of treating both hernias with a retromuscular technique. This consists of a transversus abdominis release [49], and a retromuscular Sugarbaker repair of the parastomal hernia, as described by Pauli in 2016 [50]. In this repair, one large retromuscular mesh covers both the midline and parastomal hernia. If the midline incisional hernia does not require component separation techniques, we prefer an intraperitoneal repair covering both hernia sites after closure of the defects. This involves an intraperitoneal Sugarbaker repair for the parastomal hernia. Both techniques can be performed using minimally invasive (often robotic-assisted) surgery, or by open surgery [5, 51].

Second, a cystectomy for oncological reasons involves stripping of the peritoneum below the arcuate line. This complicates extraperitoneal mesh placement in the repair of ileal conduit parastomal hernias, and therefore, intraperitoneal techniques are more convenient if no concomitant midline incisional hernia is present. Obviously, this also makes closure of the posterior layer after transversus abdominis release more difficult, and may require protecting the peritoneal cavity from mesh in the retromuscular position using omentum, biological mesh or absorbable mesh. If no midline incisional hernia is present, an intraperitoneal Sugarbaker repair of the parastomal hernia is our treatment of choice, as current evidence from surgical repairs of colostomy parastomal hernias supports the use of Sugarbaker repair [7].

A third element complicating repair of ileal conduit parastomal hernia, is that lateralization of the stomal loop (which is required to perform an adequate Sugarbaker repair) often is difficult. This is mainly due to the short mesentery of the ileal conduit loop, which is usually significantly shorter than in colostomies. In this case, often a keyhole repair is the only treatment possible [6].

Furthermore, a difficult identification of the stomal loop due to longstanding collapse, and the presence of ureteric anastomoses with the stomal loop are some other elements that complicate repair of ileal conduit parastomal hernias. Perioperative catheterization and instillation of the stomal loop may help to identify these structures during surgery.

In conclusion, repair of these specific type of hernias is considered highly complex, and the treatment of choice should depend on the presence of a midline incisional hernia, need for component separation to repair the midline incisional hernia, and the perioperative characteristics of the ileal conduit parastomal hernia (Additional file 3).

### Ongoing research and future perspectives

Besides included full text articles, 16 conference abstracts, 17 case reports, and 16 small (n < 5) case series were identified on the topic, representing a growing variety in operative techniques and mesh configurations. Upon request, two authors of the reported conference abstracts affirmed that more extensive full-text articles will follow in the near future [24, 45]. One German group shared more insights on their promising experience on the retromuscular Sugarbaker procedure, which they made their standard approach for parastomal hernia repair at the ileal conduit site [17].

Promising results of prophylactic mesh placement in end colostomies, and the specific challenges a surgical treatment of ileal conduit parastomal hernias offers, have recently raised interest in prophylactic mesh placement within this specific patient group [3]. For end colostomies there are several randomized controlled trials of good quality suggesting that placing mesh during the index operation reduces the risk of parastomal hernia while not increasing postoperative morbidity [52]. However, for urinary diversion, the evidence for prevention of parastomal hernia is as limited as literature on repair thereof. Currently there is one randomized controlled trial from Liedberg et al. They reported on a significant reduction of parastomal hernia in patients with prophylactic mesh (11% vs 23%) after 3 years of follow-up in a patient group of 181 patients [53]. Initial results seem promising, though the quality of evidence is poor [54–57]. Two other randomized trials on the topic are currently recruiting [58, 59].

## Conclusion

Generally, data on the surgical treatment of ileal conduit parastomal hernias is scarce and of poor quality. Furthermore, the absence of peritoneum below the arcuate line and a complicated identification and lateralization of the ileal conduit loop make surgical treatment of this condition complex. These limited data and perioperative challenges stress the need for prospective research on the matter including higher patient numbers. We believe that the surgical treatment of this condition requires dedicated surgical teams with adequate proficiency in this type of surgery. This systematic review does not allow to identify the optimal surgical treatment of this specific condition.

## Supplementary Information

**Additional file 1. **Study protocol. The predefined study protocol that was also registered in the PROSPERO database, reference number CRD42021226660.

**Additional file 2.** Search strategy. An outline of the systematic search strategy and results thereof.

**Additional file 3.** PRIMSA checklist. Completed checklist of PRISMA guidelines on conducting a systematic review.

## Data Availability

The predefined study protocol can be found in Additional file 1: Appendix S1, a detailed description of the literature search is added as Additional file 2: Appendix S2. No datasets were generated or analyzed during the current study.
