# Supplementary material for: Parastomal hernias after cystectomy and ileal conduit urinary diversion: surgical treatment and the use of prophylactic mesh: a systematic review
Source: BMC Surg. 2022 Mar 29;22:118. doi: 10.1186/s12893-022-01509-y (PMC8966280; doi:10.1186/s12893-022-01509-y)
Supplement: Supplementary file 1 — Additional file 1. Study protocol. The predefined study protocol that was also registered in the PROSPERO database, reference number CRD42021226660. [file 12893_2022_1509_MOESM1_ESM.pdf]

SURGICAL TREATMENT OF PARASTOMAL HERNIAS AFTER CYSTECTOMY AND ILEAL CONDUIT URINARY DIVERSION

A SYSTEMATIC REVIEW

Review Protocol

Dewulf M[1,2], Hildebrand N[1], Bouwense SAW[1], Bouvy N[1], Muysoms F[2]

[1]Department of Surgery, Maastricht UMC+, Maastricht, The Netherlands
[2]Department of Surgery, Maria Middelares, Gent, Belgium

Review protocol (30/11/2020)

For this review protocol, the Cochrane Handbook for systematic reviews and interventions was used as a guidance.

1. Title

Surgical treatment of parastomal hernias after cystectomy and ileal conduit urinary diversion: a systematic review

2. Authors

Dewulf M, Hildebrand N, Bouwense S, Bouvy N, Muysoms F

3. Online registration

This review was prospectively registered in the international prospective register of systematic reviews (PROSPERO database) on 16-12-2020.

4. Submission

The following journals will be considered for submission:

- Surgical Endoscopy
- Hernia
- Langenbeck's Archives of Surgery

5. Setting the research question

Formulating review question

With this review we aim to collect current evidence on the surgical treatment of parastomal hernias following cystectomy and ileal conduit urinary diversion. After the creation of a stoma, parastomal hernias pose a major problem regarding incidence, surgical treatment and recurrence rates. The surgical treatment of parastomal hernias has been well studied in case of colostomy, yet literature on the topic after the creation of a urinary diversion using an ileal conduit is scarce. Thereby, some specific characteristics may add significant difficulty to the surgical treatment. The absence of peritoneum after radical cystectomy with subsequent scarring of the lower abdomen, short meso complicating lateralization of the loop and the presence of ureters entering the limb are specific characteristics that make surgical repair using traditional techniques challenging.

Predefining objectives

- Participants: all patients that underwent cystectomy with ileal conduit urinary diversion
- Interventions: local surgical treatment of the parastomal hernia
- Comparators: relocation of the stoma
- Outcome: intraoperative complications, postoperative complications within 30-days after surgery (defined according to the Clavien-Dindo classification), recurrence rates and patient-reported outcome measures or quality of life scores.

Considering potential adverse effects

In order to address potential adverse effects of prehabilitation measures, non-randomized studies and qualitative research will not be excluded from this review.

Considering equity and specific populations

Studies that lack information on ASA scores, age or sex in patient groups will be excluded from the analysis. Patients under the age of 18 years will be excluded from this analysis, as the pediatric population forms a specific patient group where other techniques in the primary surgery and in the treatment of parastomal hernias may be used. No specific subgroup analyses are planned.

6. Setting the eligibility criteria for including studies in the review

Predefining unambiguous criteria for participants

Inclusion criteria

- Patients that underwent cystectomy and ileal conduit urinary diversion
- For a benign or malignant indication
- Surgery performed as open, conventional laparoscopic, robotic-assisted laparoscopic or hand-assisted procedure
-

Exclusion criteria

- Patients under the age of 18 years
- Patients that underwent cystectomy with urinary diversion using jejunal or colonic conduit, ureterostomy, ureterosigmoidostomy or orthotopic neobladder reconstruction
- Patients in which prophylactic mesh was used at the site of the ileal conduit during primary surgery

Predefining a strategy for studies with a subset of eligible participants

When only a subset of the patients is considered eligible, and data from eligible participants cannot be retrieved, authors will be contacted to provide the data. If this is not successful, studies will be excluded from analysis.

Predefining unambiguous criteria for interventions and comparators

- Criteria for interventions: local surgical treatment of the parastomal hernia using conventional laparoscopic, robotic-assisted laparoscopic or open techniques (mesh repair using an intraperitoneal Sugarbaker, retromuscular Sugarbaker, retromuscular keyhole, onlay keyhole technique or tissue repair)
- Criteria for comparators: relocation of the urinary diversion as a treatment of a parastomal hernia

Clarifying role of outcomes

Studies where the aforementioned outcomes were not the primary or secondary endpoint will not be excluded from the analysis. If the data are not available in the publication, authors will be contacted to obtain them.

Predefining study designs, including randomized trials, justifying choice of study designs

The aim of this review is to collect all evidence that is currently available. No studies will be excluded based on study design or length of follow-up. We believe that this addresses the aim of this review and reduces potential publication bias. Case reports, animal studies and case series reporting on less than 5 patients will be excluded from the analysis.

Excluding studies based on publication

Studies will be included irrespective of the publication status. Efforts will be made to obtain and include data from unpublished and ongoing trials by contacting authors.

Changing eligibility criteria

Any changes to eligibility criteria and to the protocol will be mentioned and justified in the paper.

7. Selecting outcomes to be addressed for studies included in the review

Predefining outcome domains – choosing outcomes – predefining outcome measures

The following outcomes are defined.

Primary endpoint:

- Postoperative complications within 30 days after surgery (defined according to the Clavien-Dindo classification)

Secondary endpoint:

- Recurrence rates, diagnosed by clinical examination of radiological evaluation

Furthermore, data on the following endpoints will be collected, when available:

- Intraoperative complications
- Patient-reported outcome measures
- Quality of life scores

8. Performing the review – searching for studies

Searching general bibliographic databases and CENTRAL

For the literature search CENTRAL (Cochrane Central Register of Controlled Trials, MEDLINE (through PubMed), Web of Science, and Embase will be searched. Duplicates will be identified and removed using Endnote software (Clavirate Analytics, Philadelphia, US). Results will be screened by abstract, and subsequently by evaluation of full text. When no full text is available, authors will be contacted. No restrictions regarding language will be applied. The search will be independently conducted by the two first authors. In case of discrepancy, the senior author will be consulted.

Searching specialist bibliographic databases – grey literature

Abstract books of the most important meetings in the field (annual meetings of the European Hernia Society, European Association of Endoscopic Surgery and American Hernia Society) of the last 2 years will be searched for the topic to identify additional sources.

Searching for different types of evidence

As there are no restrictions regarding types of evidence, no separate search strategies will be undertaken.

Searching trials registers

Both ClinicalTrials.gov and the International Clinical Trials Registry Platform (ICTRP) portal will be searched to identify ongoing studies on the topic. In case of relevant ongoing studies, authors will be contacted to specify the nature of the ongoing research and, when available, preliminary results.

Searching within other reviews

Other reviews on the topic will be evaluated to identify additional sources.

Searching reference lists

Reference lists of included studies will be checked to identify additional sources.

Structuring search strategies for bibliographic databases - Developing search strategies for bibliographic databases

The following search terms will be used, using the Boolean operators ‘OR’ and ‘AND’:

- Cystectomy OR
- Urinary diversion OR
- Ileal conduit OR
- Urostomy

AND

- Hernia OR
- Parastomal hernia

This eventually results in the following search:
(((((cystectomy) OR urinary diversion) OR ileal conduit) OR urostomy)) AND ((hernia) OR parastomal hernia)

Using search filters

In order to avoid missing important sources, no specific search filters will be used when conducting the search.

Restricting database searches

No date restrictions or publications format restrictions will be used. Case reports, animal studies and case series reporting on less than 5 patients will be excluded from the analysis.

Documenting the search process

Details on the search process will be added as an addendum to the publication, in a way that is reproducible at all times.

Rerunning searches – incorporating finding from rerun searches

The search will be rerun close to publication, with a maximum of 6 months from the intended publication date. Additional studies will be fully incorporated in the review.

Assessment of methodological quality

The methodological quality of included studies was independently assessed by the two first authors (MD and NH). For randomized controlled trials (RCTs), the Cochrane Collaboration's tool for assessing risk of bias was used. Selection, performance, detection, attribution, reporting and overall bias were reported as 'low risk' (green), 'high risk' (red) or 'unclear' (yellow). Non-randomized trials were scored using the ROBINS-I tool. Risk of bias was defined as 'low' (green), 'moderate' (yellow) or 'serious' (red). In case of discrepancy, the risk of bias was discussed with the senior author until consensus was reached.
