# Supplementary material for: Parastomal hernias after cystectomy and ileal conduit urinary diversion: surgical treatment and the use of prophylactic mesh: a systematic review
Source: BMC Surg. 2022 Mar 29;22:118. doi: 10.1186/s12893-022-01509-y (PMC8966280; doi:10.1186/s12893-022-01509-y)
Supplement: Supplementary file 2 — Additional file 2. Search strategy. An outline of the systematic search strategy and results thereof. [file 12893_2022_1509_MOESM2_ESM.pdf]

Surgical Treatment of Parastomal Hernias after Cystectomy and Ileal Conduit Urinary Diversion – A systematic review

Hernia, Dewulf M, Hildebrand ND, Bouwense SAW, Boucy ND, Muysoms F
Maastricht UMC+, Maria Middelares Gent
Corresponding author: dewulfmaxime@gmail.com

Appendix 2- Search strategy

Search in detail:

PubMed

17.12.2020:

((((((abdominal hernia[MeSH Terms]) OR (hernia[MeSH Terms])) OR (hernia)) OR (abdominal hernia)) OR (parastomal hernia[MeSH Terms])) OR (parastomal hernia)) AND ((((((((cystectomy[MeSH Terms]) OR (cystectomy)) OR (urinary diversion[MeSH Terms])) OR (urinary diversion)) OR (ileal conduit[MeSH Terms])) OR (ileal conduit))) OR (urostomy))

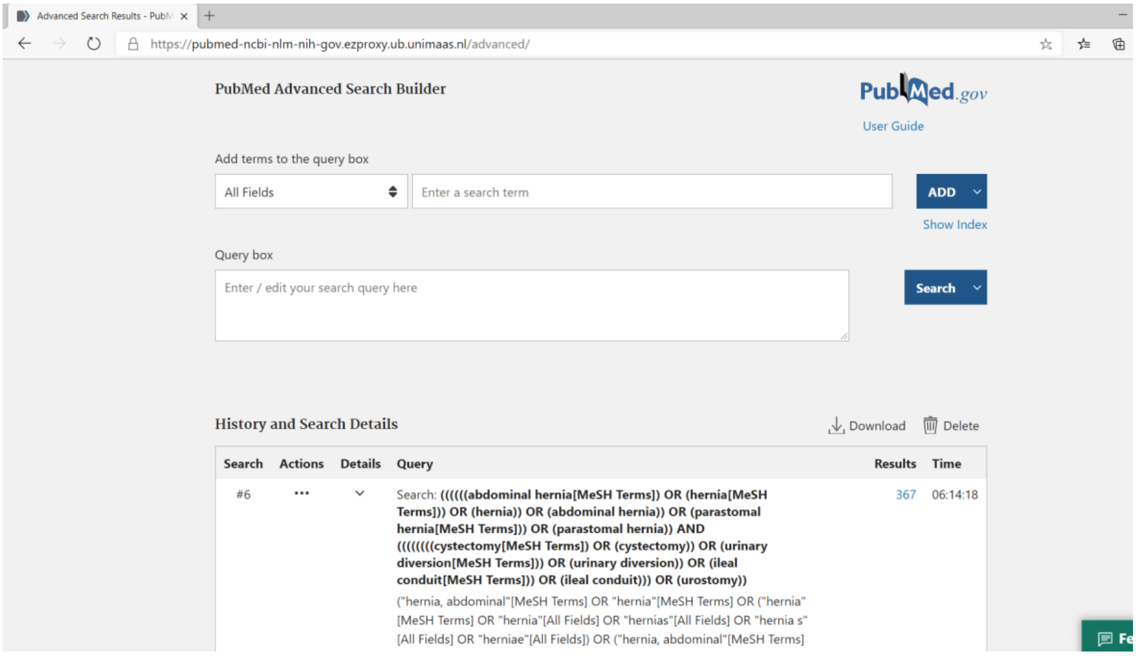

(((((cystectomy) OR urinary diversion) OR ileal conduit) OR urostomy)) AND ((hernia) OR parastomal hernia)

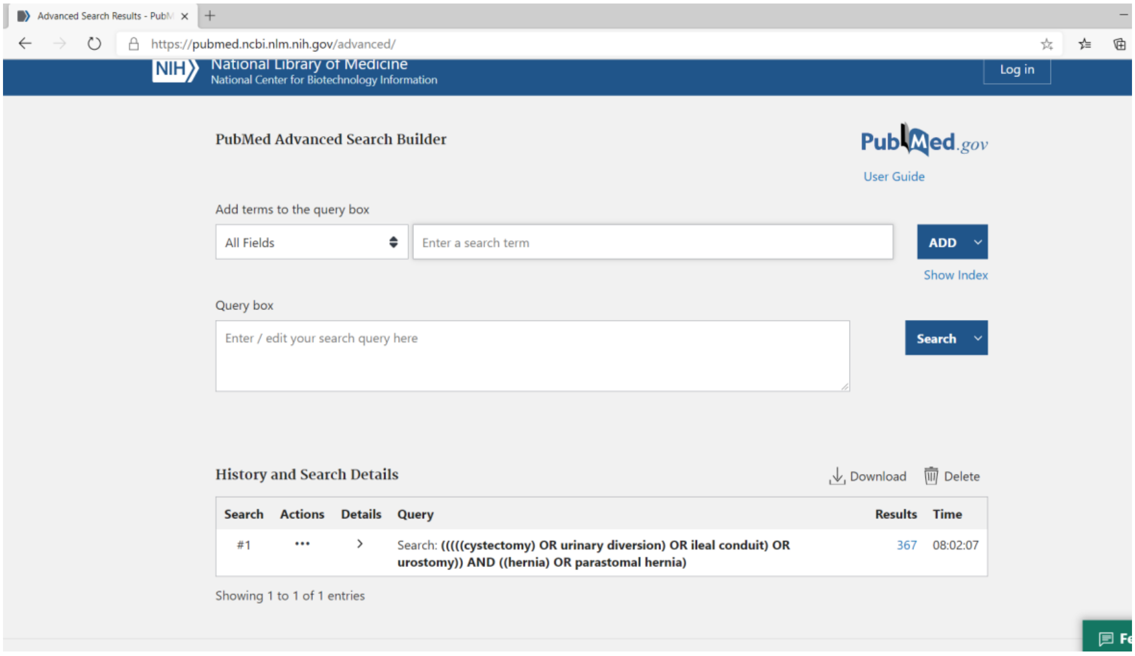

Results: 367

Imported to Endnote: 367

After removal of duplicates: 356

Embase:

17.12.2020

((cystectomy or urinary diversion or ileal conduit or urostomy) and parastomal hernia).af.

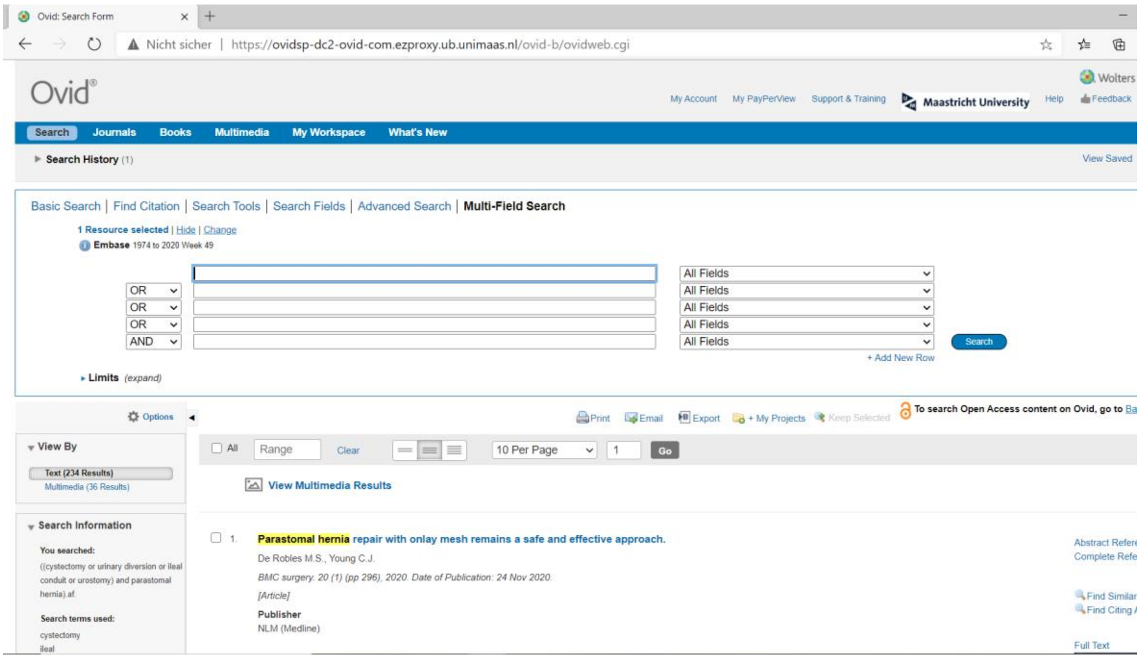

Results: 234

Updated to Endnote: 234

After removal duplicates: 128

Web of Science:

17.12.2020

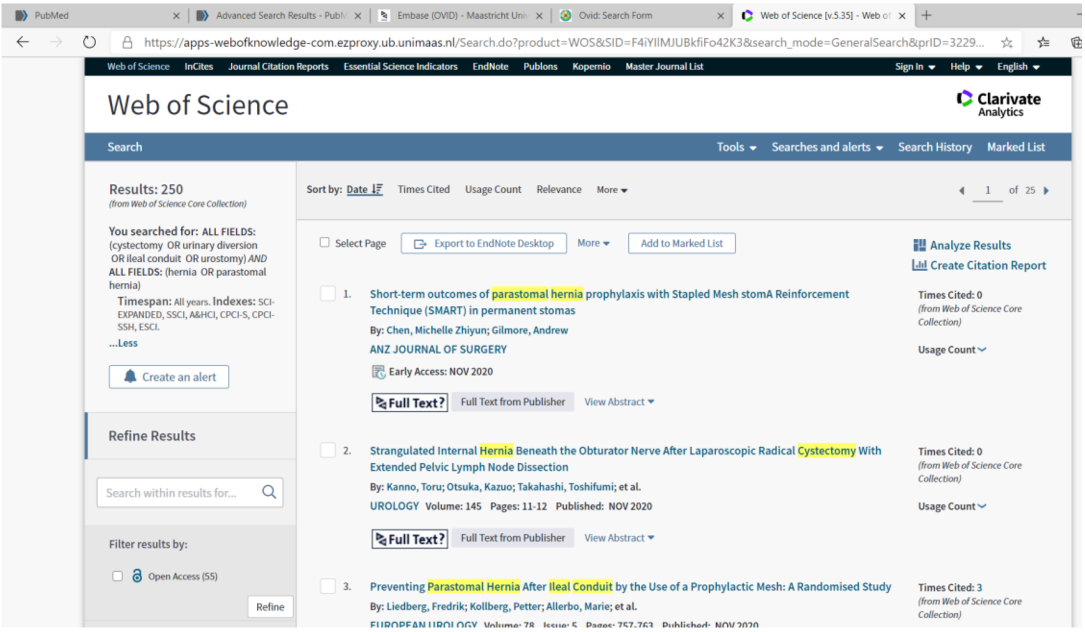

Results: 250

Imported to Endnote: 250

After removal of duplicates: 58

ALL FIELDS: (cystectomy OR urinary diversion OR ileal conduit OR urostomy) AND ALL FIELDS: (hernia OR parastomal hernia)

CENTRAL database:

18.12.2020

(cystectomy OR urinary deviation OR ileal conduit OR urostomy) in Title Abstract Keyword AND parastomal hernia OR hernia in Title Abstract Keyword - (Word variations have been searched)

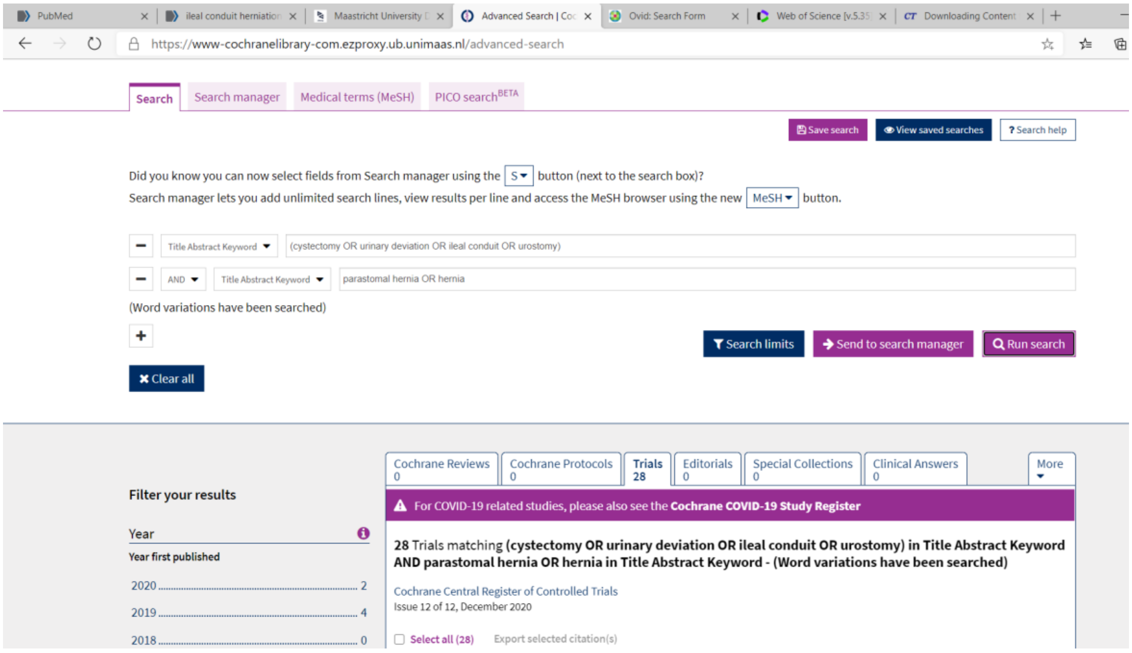

Results: 0 reviews, 28 trials

Imported to Endnote: 28

After removal of duplicates: 26

Clinicaltrials.gov

18.12.2020

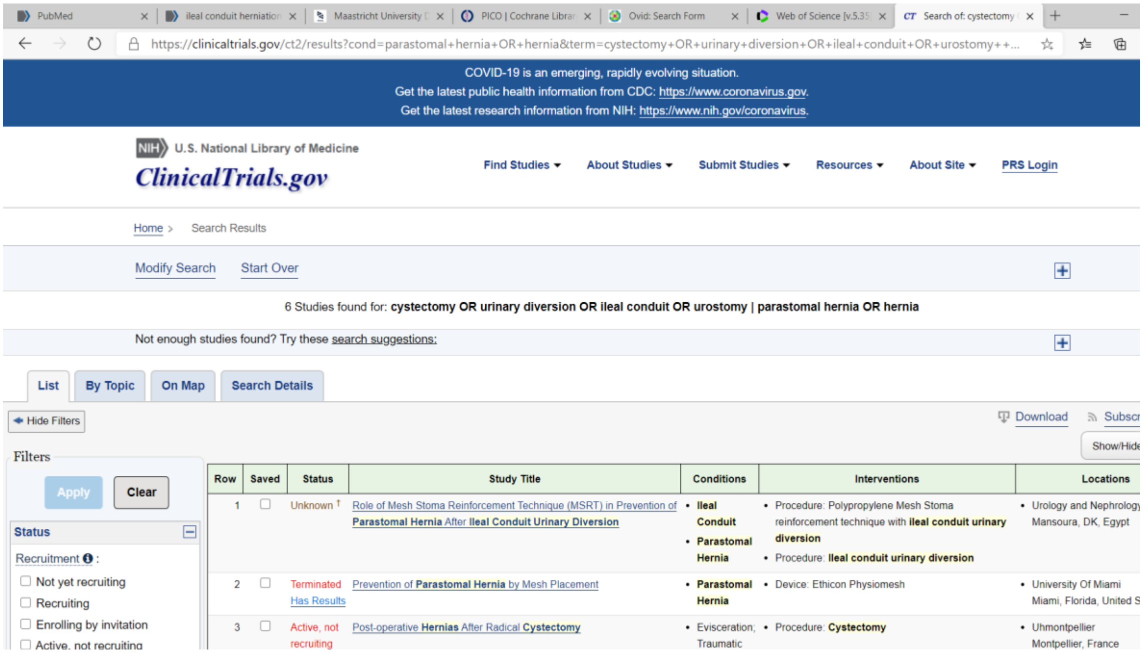

Results: 6 trials

Imported to Endnote: 6

After removal of duplications: 6

Additional sources:

- European hernia society: Antoniou SA, Agresta F, Garcia Alamino JM et al (2018) European Hernia Society guidelines on prevention and treatment of parastomal hernias. Hernia 22:183–198.
- European hernia society: Congres abstracts 2019 & 2020: 2 abstract with subgroups -> sent e-mails: no additional data available
    - Laparoscopic treatment of parastomal hernias: a single center experience with the Pauli procedure M. Borshchigov, C. Gröger, e.a.
    - Parostomal hernias: a more and more surgical challenge J. A. Martín
- American hernia society: Congres abstracts 2019 & 2020: 1 abstract -> sent e-mail: article will follow soon
    - KEYHOLE VERSUS SUGARBAKER TECHNIQUES IN PARASTOMAL HERNIA REPAIR AT BRICKER CONDUIT: A RETROSPECTIVE NATIONWIDE COHORT STUDY WITH LONG-TERM FOLLOW-UP Elisa Mäkäräinen-Uhlbäck, MD ; Jaana Vironen, MD, PhD
- Systematic reviews:
    - Narang SK, Alam NN, Campain NJ, Pathak S, McGrath JS, Daniels IR, et al. Parastomal hernia following cystectomy and ileal conduit urinary diversion: a systematic review. Hernia. 2017;21(2):163-75.
    - DeAsis FJ, Lapin B, Gitelis ME, Ujiki MB (2015) Current state of laparoscopic parastomal hernia repair: a meta-analysis. World J Gastroenterol 21:8670–8677
    - Hansson BME, Slater NJ, Van der Velden AS, et al. Surgical techniques for parastomal hernia repair: a systematic review of the literature. Ann Surg. 2012;255(4):685–95.
    - Aquina CT, Iannuzzi JC, Probst CP, Kelly KN, Noyes K, Fleming FJ, Monson JR. Parastomal hernia: a growing problem with new solutions. Dig Surg. 2014;31(4-5):366-76. doi: 10.1159/000369279. Epub 2014 Dec 13. PMID: 25531238.

Screening by title:

Results in Endnote: 881

After removal of duplicates: 571+7 added based on references +3 from congress abstracts

After Screening by title: 130

Screening by abstract:

Results in Endnote: 127 (+3 conference abstracts)

After screening by abstract: 42 (+3 conference abstracts)

**Author**	**Inclusion**	**Reason for exclusion**	**References checked**
Abaza 2015	No after abstract	Inadequate study design	N/A
Abdallah 2009	No after abstract	Not matching inclusion criteria	N/A
Aldaahm 2019	Yes	Robot-assisted repair, 7 cases: conference abstract	None
Amimi 2015	No after abstract	Not machting inclsusion criteria	N/A
Ando 2020	No after abstract	Insufficient patient number	N/A
Antor 2017	Yes	Laparoscopic repair, 9 cases: conference abstract	None
Avallone 2018	No after abstract	Not matching inclusion criteria	N/A
Ayuso 2020	Yes	Robot-assisted repair, 5 cases urostomy -	DeAsis 2015
Berger 2007	Yes	Laparoscopic repair, 66 patients; subgroup?	None
Berrevoet 2018	No after abstract	Insufficient patient number	N/A
Bos 1996	Yes	Not available?	None
Bosanquet 2015	No after abstract	Inadequate study design	N/A
Castillo 2006	No after abstract	Not matching inclusion criteria	N/A
Craft 2008	Yes	Laparoscopic repair, 9 cases -> subgroup, older article, mixed techniques (keyhole and sugarbaker)	Steele 2003
Davis 2012	Yes	Laparoscopic repair, 11 cases; conference abstract, technique not specified	None
De Robles 2020	Yes	Mesh repair vs relocation, 16 cases (subgroup); both techniques combined in results	None
Deol 2003	No after abstract	Inadequate study design	N/A
Doddamani 2015	No after abstract	Not matching inclusion criteria	N/A
Donahue 2016	No after abstract	Not matching inclusion criteria	N/A
Dunet 2002	No after abstract	Inadequate study design	N/A
Elkarta 2020	No after abstract	Not matching inclusion criteria	N/A
Faba 2009	Yes	Conference abstract, different techniques, 28 cases	None
Faba 2011	Yes	Open repair, 19 cases	None
Farnham 2004	No after abstract	Not matching inclusion criteria	N/A

Fei 2011	No after abstract	Insufficient patient number	N/A
Fischer 2017	Yes	3 mesh repair, 9 cases (subgroup?)	None
Fitzgerald 2017	Yes	Top hat repair, 22 cases	Smarts 2011
Franks 2001	Yes	Keyhole technique, 6 cases	None
Gadissa 2010	No after abstract	Inadequate study design	N/A
Ganga 2017	No after abstract	Inadequate study design	N/A
Garcia Rojo 2016	No after abstract	Inadequate study design	N/A
Gilbert 2013	No after abstract	Not matching inclusion criteria	N/A
Gosselink 2015	No after abstract	Inadequate study design	N/A
Gould 2003	No after abstract	Inadequate study design	N/A
Gupta 2020	Yes	Robotic sugarbaker repair, 5 cases	None
Hansson 2013	No after abstract	Insufficient patient numbers	N/A
Helal 1997	Yes	Mesh repair, 19 cases	
Hetet 2005	No after abstract	Not matching inclusion criteria	N/A
Ho 2004	Yes	Lateral approach repair, 15 cases	None
Holmes 2002	No after abstract	Not matching inclusion criteria	N/A
Hopkins 1982	No after abstract	Inadequate study design	N/A
Husain 2008	No after abstract	Inadequate study design	N/A
Hussein 2017	Yes	Robot vs open, 6 cases	None
Hussein 2016	No after abstract	Not matching inclusion criteria	N/A
Jaipura 2020	Yes	Robotic sugarbaker repair, 6 cases; conference abstract	None
Jeekel 2017	Yes	Conservatively vs surgery, 6 cases (subgroup)	None
Kasperk 2000	No after abstract	Not matching inclusion criteria	N/A
Kaufman 1983	No after abstract	Insufficient patient numbers	N/A
Khanna 2012	No after abstract	Not matching inclusion criteria	N/A
Knap 2004	No after abstract	Not matching inclusion criteria	N/A
Kotes 2018	Yes	Open with mesh, 23 patients; subgroup + conference abstract	None

Kouba 2007	No after abstract	Not matching inclusion criteria	N/A
Kozlowski 2001	No after abstract	Insufficient patient numbers	N/A
Kroese 2018	Yes	Conservative vs surgery (subgroup?)	None
Lambrichts 2017	Yes	Conference abstract; conservative vs surgery, 8 cases (subgroup)	None
Lampel 2012	Yes	Intraperitoneal slit mesh (new kind of mesh), ? cases	None
LeBlanc 2005	No after abstract	Insufficient patient numbers	N/A
Lee 2018	No after abstract	Inadequate study design	N/A
Li 2019	No after abstract	Not matching inclusion criteria	N/A
Liard 2001	No after abstract	Not matching inclusion criteria	N/A
Lima 2010	No after abstract	Inadequate study design	N/A
Maidaa 2014	No after abstract	Not matching inclusion criteria	N/A
Majumder 2016	No after abstract	Insufficient patient numbers	N/A
Mäkelä 1997	No after abstract	Not matching inclusion criteria	N/A
Malak 2017	No after abstract	Inadequate study design	N/A
Marshall 1975	Yes	Translocation?	None
McAllister 2018	No after abstract	Inadequate study design	N/A
McLemore 2007	Yes	Conventional vs laparoscopic, 19 cases (subgroup?)	Deol 2008
Mekhail 2017	No after abstract	Insufficient patient numbers	N/A
Mekhail 2017	No after abstract	Inadequate study design	N/A
Miller 2016	Yes	Retromuscular repair, 19 cases, conference abstract	None
Mirza 2008	No after abstract	Insufficient patient numbers	N/A
Mitchell 2011	No after abstract	Insufficient patient numbers	N/A
Mizrahi 2011	No after abstract	Insufficient patient numbers	N/A
Mizrahi 2012	No after abstract	Insufficient patient numbers	N/A
Movassaghi 2016	No after abstract	Not matching inclusion criteria	N/A
Myers 2016	No after abstract	Inadequate study design	N/A

Nomura 2003	No after abstract	Inadequate study design	N/A
Olmi 2019	Yes	Keyhole repair, 7 cases (subgroup?)	None
Oma 2017	No after abstract	Not matching inclusion criteria	N/A
Palanivelu 2017	No after abstract	Insufficient patient numbers	N/A
Pfister 2020	No after abstract	Not matching inclusion criteria	N/A
Raigani 2014	Yes	Relocation/mesh repair, 10 cases (subgroup?)	Rosen 2010
Rajapandian 2020	Yes	Laparoscopic keyhole repair, subgroup?	None
Ramalingam 2013	No after abstract	Inadequate study design	N/A
Ramalingam 2014	No after abstract	Inadequate study design	N/A
Rege 2019	No after abstract	Insufficient patient numbers	N/A
Rezaee 2020	No after abstract	Not matching inclusion criteria	N/A
Rodriguez 2009	No after abstract	Duplication	N/A
Rodriguez 2011	No after abstract	Duplication	N/A
Roghmann 2015	No after abstract	Not matching inclusion criteria	N/A
Romo 2015	No after abstract	Inadequate study design	N/A
Rosen 2010	No after abstract	Not matching inclusion criteria	N/A
Safadi 2004	Yes	Laparoscopic repair, 5 cases (subgroup?)	None
Sanseverino 2002	Yes	New technique, ? cases	None
Sanseverino 2002	No after abstract	Duplication	N/A
Shakir 2020	Yes	Robotic mesh repair, 7 cases; conference abstract	None
Sharma ?	Yes	Repair in general, 23 cases; conference abstract	None
Simeone 2003	No after abstract	Not matching inclusion criteria	N/A
Singh 2020	No after abstract	Inadequate study design	N/A
Smart 2011	No after abstract	Not matching inclusion criteria	N/A
Steele 2003	No after abstract	Not matching inclusion criteria	N/A
Su 2020	No after abstract	Not matching inclusion criteria	N/A
Sunaoshi 2003	No after abstract	Inadequate study design	N/A

Suwa 2016	No after abstract	Insufficient patient numbers	N/A
Syan 2012	No after abstract	Not matching inclusion criteria	N/A
Talab 2018	No after abstract	Inadequate study design	N/A
Tan 2017	No after abstract	Inadequate study design	N/A
Tatay 2017	Yes	New technique, 6 cases (subgroup?)	None
Trunbull 2003	No after abstract	Inadequate study design	N/A
Tully 2017	Yes	Open repair, 27-40 cases; conference abstract	None
Tully 2019	Yes	Open repair, 27-40 cases	Duplication?
Van Ginkel 2019	No after abstract	Not matching inclusion criteria	N/A
Velazco 2016	Yes	Different techniques, 16 cases (subgroup?)	None
Von Bodman 2012	Yes	Mesh repair, 13 cases	None
Von Rundstedt 2014	No after abstract	Insufficient patient numbers	N/A
Wara 2016	No after abstract	Not matching inclusion criteria	N/A
Warren 2015	Yes	Retromuscular repair, 6 patients (subgroup?); conference abstract	None
Wood 2004	No after abstract	Not matching inclusion criteria	N/A
Yang 2017	No after abstract	Inadequate study design	N/A
Ypsilantis 2015	Yes	Sugarbaker repair, 15 cases (subgroup?)	None

Screening by full text:
Results in Endnote: 42 (+ 3 conference abstracts)
After screening by full text: 6 (+conference abstracts/subgroup analyses)

(1) **Author**: Aldhaam NA, Elsayed AS 2019
Title: Minimally invasive management of robot-assisted radical cystectomy related complications: 15 years of experience
Journal: Journal of Endurology, *Conference abstract*: 37th World Congress of Endurology, Abu Dhabi
Patients: patients undergoing robot-assisted repair of cystectomy complications; *subgroup* of 7 patients with parastomal hernia
Intervention: Robot-assisted repair of any kind of complication requiring surgical repair after radical cystectomy
Clinical outcome: Need of surgical repair

E-mailed, no response.
EXCLUSION: conference abstract + subgroup: no additional/specific data available
(not matching inclusion criteria)

(2) **Author**: Antor M, Schwarz L 2017
Title: Laparoscopic repair of parastomal hernia after radical cystectomy
Journal: Hernia; *conference abstract*: 39th Annual International Congress of the European Hernia Society, Prevention and Prophylaxis beyond Hernia Surgery. Austria
Patients: 9 patients undergoing laparoscopic repair for symptomatic parastomal hernia of ileal conduit
Intervention: Laparoscopic repair of urostomy incisional hernia; technique not specified; no complications occurred.
Clinical outcome: postoperative complications short-term and long-term, hospital stay operation time, recurrence

E-mail not available.
EXCLUSION: conference abstract; no additional data available
(not matching inclusion criteria)

(3) **Author:** Ayuso SA, Shao, JM 2020
Title: Robotic sugarbaker parastomal hernia repair: technique and outcomes
Journal: Hernia
Patients: patients requiring surgical repair for parastomal hernia, *subgroup* of 5 urostomy patients
Intervention: Robotic sugarbaker repair of parastomal hernia's;
Clinical outcome: operative time, intra-operative complications, length of stay, postoperative complications, and recurrence

E-mailed, no response
EXCLUSION: subgroup; no specific data available
(not matching inclusion criteria)

(4) **Author:** Berger D, Bientzle M, 2007
Title: Laparoscopic repair of parastomal hernias: a single surgeon's experience in 66 patients
Patients: patients undergoing laparoscopic repair in a single center; 1 ileal conduit patient
Intervention: laparoscopic onlay mesh (sometimes 2 meshes)
Clinical outcome: recurrence

EXCLUSION: insufficient patient number

(5) **Author:** Bos SD
Title: Incidence and therapy of parastomal hernia in urology
Journal: Nederlands Tijdschrift voor Geneeskunde

EXCLUSION: review (inadequate study design)

(6) **Author:** Craft RO, Huguet KL 2008
Title: Laparoscopic parastomal hernia repair

Journal: Hernia
Patients: patients undergoing laparoscopic repair of parastomal hernias; *subgroup* of 9 patients with ileal conduit
Intervention: sugarbaker or keyhole laparoscopic parastomal hernia repair
Clinical outcome: operative time, intra-operative complications, length of stay, postoperative complications (not stated how fast after surgery), recurrence

E-mail, no response.
EXCLUSION: subgroup, no specific data available
(not matching inclusion criteria)

(7) **Author:** Davis PJ, Klassen DR 2012
Title: Laparoscopic repair of parastomal hernias of ileal conduits
Journal Surgical Endoscopy and Other Interventional Techniques; *Conference abstract*: 2012 Scientific Session of the Society of American Gastrointestinal and Endoscopic Surgeons (SAGES)
Patients: patients undergoing attempted laparoscopic repair of a ileal conduit parastomal hernia
Intervention: laparoscopic parastomal hernia repair, technique not specified
Outcome: operative time, blood loss, length of stay, postoperative complications (none are described), recurrence

E-mail, dataset not available anymore.
EXCLUSION: conference abstract; no additional data available
(not matching inclusion criteria)

(8) **Author:** De Robles MS, Young CJ 2020
Title: Parastomal hernia repair with onlay mesh remains a safe and effective approach
Journal: BMC Surg
Patients: patients undergoing relocation or mesh repair for symptomatic parastomal hernia, *subgroup* of 16 patients with ileal conduit
Intervention: either open relocation of mesh repair with peristomal or midline incision
Outcome: mesh related complications, recurrence, operative time, length of stay

E-mail, no response.
EXCLUSION: subgroup; no specific data available
(not matching inclusion criteria)

(9) **Author:** Faba OR, Rosales, A 2011
Title: Simplified technique for parastomal hernia repair after radical cystectomy and ileal conduit creation
Journal: Urology
Patients: patients requiring surgical repair of ileal conduit parastomal hernia, 19 patients
Intervention: ipsilateral relocation of the stoma
Outcome: operative time, length of stay, postoperative complications, recurrence

INCLUSION

(10)**Author:** Faba OR, Rosales A, 2009
Title: Post-radical cystectomy parastomal hernia repair: long-term follow-up
Journal: European Urology Supplements; *conference abstract*: 24th Annual Congress of the European Association of Urology, Stockholm, Sweden
Patients: patients requiring surgical repair of ileal conduit parastomal hernia
Intervention: 19 patients ipsilateral relocation, 9 patients local fascia repair
Outcome: postoperative complications, operative time, recurrence

EXCLUSION: duplication (e-mail authors for 9 additional patients, no response)

(11)**Author:** Fischer I, Wundsam H, 2017
Title: Parastomal hernia repair with a 3D funnel intraperitoneal mesh device and same-sided stoma relocation: results of 56 cases
Journal: World journal of surgery
Patients: patients undergoing laparoscopic repair of parastomal hernia; *subgroup* with 7 ileal conduit patients
Intervention: Laparoscopic repair with mesh
Outcome: surgical complications, recurrence and treatment thereof

E-Mail, no response
EXCLUSION: subgroup; no specific data available
(not matching inclusion criteria)

(12)**Author:** Fitzgerald MJ, Ulrich S 2018
Title: Parastomal hernia repair using the "top hat" technique - An initial experience in 30 patients at Memorial Sloan Kettering Cancer Center
Journal: American Journal of Surgery
Patients: patients undergoing top-hat repair of a parastomal hernia; *subgroup* with 22 ileal conduit patients
Intervention: open top-hat repair of parastomal hernia
Outcome: operative time, postoperative complications, recurrence

E-Mail, no response.
EXCLUSION: subgroup, no specific data available
(not matching inclusion criteria)

(13)**Author:** Franks ME, Hrebinko RL, 2001
Title: Technique of parastomal hernia repair using synthetic mesh
Journal: Urology
Patients: 6 patients with ileal conduit parastomal hernia undergoing mesh repair
Intervention: supraperitoneal fascial closure and mesh repair
Outcome: recurrence, ‘no infections’, ‘no stenoses’

INCLUSION

(14)**Author:** Gupta S, Rawal S, 2020
Title: Robotic intraperitoneal onlay graft hernioplasty for ileal conduit urinary diversion associated parastomal hernia

Journal: Journal of Urology; *conference abstract*: 2020 Annual Meeting of the American Urological Association. United States.
Patients: patients requiring surgical repair of ileal conduit parastomal hernia
Intervention: robotic intraperitoneal mesh repair
Outcome: postoperative complications Clavien Dindo >2, operative time, length of stay

EXCLUSION: duplication (same as Jaipura 2020)

(15)**Author:** Helal M, Austin P, 1997
Title: Evaluation and management of parastomal hernia in association with continent urinary diversion
Journal: J Urol
Patients: 22 patients with symptomatic ileal conduit parastomal hernia
Intervention: transabdominal approach with closure of fascia and reposition of stoma
Outcome: recurrence, effect on pre-operative symptoms

INCLUSION

(16)**Author:** Ho KM, Fawcett DP 2004
Title: Parastomal hernia repair using the lateral approach
Journal: BJU Int
Patients: 15 patients with symptomatic ileal conduit associated parastomal hernia
Intervention: lateral approach, closure of the defect, covered with mesh
Outcome: recurrence

INCLUSION

(17)**Author:** Hussein A, Kozlowski J 2017
Title: Revisiting the abdomen after robot-assisted radical cystectomy: Tips and tricks for robot-assisted repair
Journal: Journal of urology; *conference abstract*: 112th Annual Meeting of the American Urological Association, AUA 2017. United States.
Patients: patients undergoing robotic/open repair of late post-operative radical cystectomy complications; *subgroup* of 4 vs 2 (robotic vs open) patients for ileal conduit parastomal hernia repair
Intervention: open vs robotic repair, not specified
Outcome: failure of treatment?

E-mail, no response
EXCLUSION: conference abstract + subgroup: no additional/specific data available
(not matching inclusion criteria)

(18)**Author:** Hussein A, Hashmi Z 2016
Title: Reoperations following robot-assisted radical cystectomy: A decade of experience
Journal: Journal of urology
Patients: all patients undergoing surgical intervention after robotic assisted radical cystectomy, *subgroup* of only 4 patients with parastomal hernia
Intervention: robotic-assisted mesh hernioplasty

Outcome: postoperative complications, time to operation, predictive factors of need for surgical intervention, hospital stay, blood transfusion

EXCLUSION: insufficient patient number

(19)**Author:** Jaipura J, Rawal SK 2020
Title: Robotic modified sugarbaker intraperitoneal onlay graft hernioplasty for ileal conduit urinary diversion associated with parastomal hernia
Journal: European Urology Open Science; *Conference abstract*: Urology Open Science. Conference: EAU20 Virtual Congress and Theme Week
Patients: patients requiring surgical repair of ileal conduit parastomal hernia
Intervention: robotic intraperitoneal mesh repair
Outcome: postoperative complications Clavien Dindo >2, operative time, length of stay

E-Mail, no additional data available.
EXCLUSION: conference abstract; no additional data available.
(not matching inclusion criteria)

(20)**Author:** Jeekel J, Landin S 2017
Title: Surgical treatment versus watchful waiting in patients with parastomal hernia: A retrospective cohort study
Journal: Hernia; conference abstract: 18th Annual Hernia Repair. Mexico.
Patients: patients with parastomal hernia with any kind of stoma; 6 ileal conduit patients
Intervention: surgical treatment, versus watchful waiting
Outcome: symptoms, crossover rates, recurrence, postoperative complications, ER-admission

EXCLUSION: duplication (Lambrichts 2017 and Kroese 2018)

(21)**Author:** Kotes S, Greenwell T 2018
Title: Is obstruction of ileal conduit after parastomal hernia repair with porcine direved tissue matrix Stratice TM a valid concern?
Journal: Journal of Clinical Urology; *conference abstract*: 2018 Annual Scientific Meeting of the British Association of Urological Surgeons, BAUS 2018. United Kingdom
Patients: patients with incisional or parastomal hernia after ileal conduit, *subgroup* of 23 patients with ileal conduit parastomal hernia
Intervention: open repair with lateral mesh fixation
Outcome: mesh-related complications, stoma related complications (obstruction), recurrence

E-mail, no response
EXCLUSION: conference abstract + subgroup: no additional/specific data available
(not matching inclusion criteria)

(22)**Author:** Kroese LF, Lambrichts DPV, 2018
Title: Non-operative treatment as a strategy for patients with parastomal hernia: a multicentre, retrospective cohort study
Journal: Colorectal Dis

Patients: patient with parastomal hernia with any kind of stoma; just 4 ileal conduit patients within the surgery treatment group
Intervention: surgical treatment, versus watchful waiting
Outcome: symptoms, crossover rates, recurrence, postoperative complications, ER-admission

E-Mail, no response
EXCLUSION: subgroup, no specific data available.
(not matching inclusion criteria)

(23)**Author:** Lambrichts DPV, Kroese LF 2017
Title: Surgical treatment versus watchful waiting in patients with parastomal hernia: A retrospective cohort study
Journal: Hernia; conference abstract: 39th Annual International Congress of the European Hernia Society, Prevention and Prophylaxis beyond Hernia Surgery. Austria
Patients: patient with parastomal hernia with any kind of stoma; 8 ileal conduit patients
Intervention: surgical treatment, versus watchful waiting
Outcome: symptoms, crossover rates, recurrence, postoperative complications, ER-admission

EXCLUSION: duplication (Jeekel 2017 and Kroese 2018)

(24)**Author:** Lampel A, Runkel N 2012
Title: Correction of parastomal hernia using meshes
Journal: Urologe A
Patients: patients requiring surgical repair of parastomal hernia after ileal conduit, n=3
Intervention: open repair with sublay mesh placement
Outcome: recurrence

EXCLUSION: insufficient patient numbers

(25)**Author:** Marshall FF, Leadbetter WF 1975

EXCLUSION: not available online -> not matching inclusion criteria

(26)**Author:** McLemore EC, Harold KL, 2007
Title: Parastomal hernia: short-term outcome after laparoscopic and conventional repairs
Journal: Surg Innov;
Patients: all patients requiring parastomal hernia repair; *subgroup* of 17 ileal conduit patients
Intervention: open (primary suture, mesh, relocation) or laparoscopic (sugarbaker, keyhole) repair of any kind of symptomatic parastomal hernia
Outcome: postoperative complications, operative time, recurrence

EXCLUSION: duplication (subgroup), same as No 6

(27)**Author:** Miller HJ, Majumder A, 2016
Title: Retromuscular repair of parastomal hernias in the setting of ileal conduit: outcomes

and special considerations
Journal: Journal of the American college of surgeons; *conference abstract* 2nd Owen H Wangensteen Scientific Forum. United States
Patients: patients who underwent retromuscular repair of parastomal hernias of ileal conduit; 19 patients
Intervention: retromuscular repair with mesh
Outcome: postoperative complications, mesh related complications, recurrence

E-mail not available.
EXCLUSION: conference abstract; no additional data available
(not matching inclusion criteria)

(28)**Author:** Olmi S, Oldani a 2019
Title: Laparoscopic modified keyhole technique with coated polyester mesh for treatment of parastomal hernia: measures for improving the outcome
Journal: J Laparoendosc Adv Surg Tech A
Patients: patients requiring surgical repair for parastomal hernia, *subgroup* of 9 ileal conduits
Intervention: keyhole technique with parietex or physiomesh
Outcome: postoperative complications, recurrence, mesh related complications

E-Mail, no response.
EXCLUSION: subgroup; no specific data available
(not matching inclusion criteria)

(29)**Author:** Raigani S, Criss CN 2014
Title: Single-Center Experience With Parastomal Hernia Repair Using Retromuscular Mesh Placement
Journal: Journal of Gastrointestinal Journal
Patients: all patients requiring repair for parastomal hernia at a single center ; subgroup of 10 ileal conduit patients
Intervention: retromuscular approach utilizing a posterior component separation with relocation to contralateral abdominal wall
Outcome: postoperative wound complications, length of follow-up and hernia recurrence

E-Mail, no response
EXCLUSION: subgroup; no additional data available
(not matching inclusion criteria)

(30)**Author:** Rajapandian S, Jankar SV, 2020
Title: Modified laparoscopic keyhole plus repair to manage a parastomal hernia: A single-center experience.
Journal: Asian J Endosc Surg
Patients: patients with symptomatic parastomal hernia requiring repair, 3 ileal conduits
Intervention: laparoscopic modified keyhole repair
Outcome: perioperative outcomes such as operative time, hospital stay, postoperative complications, recurrence

EXCLUSION: insufficient patient numbers

(31)**Author:** Safadi 2004
Title: Laparoscopic repair of parastomal hernia: early results
Jourrnal: Surg Endosc
Patients: patients with symptomatic parastomal hernia of any stoma, 5 ileal conduit
Intervention: laparoscopic mesh repair
Outcome: postoperative complications, recurrence, operative time

INCLUSION: patient details are given for all 5 ileal conduit patients

(32)**Author:** Sanseverino RC, Zucco F
Title: Marlex of Prolene mesh repair of parastomal hernia in a patient with Bricker urinary diversion
Patients: 4 patients undergoing parastomal hernia repair
Intervention: semicircular incision around stoma with interposition of mesh

EXCLUSION: insufficient patient numbers

(33)**Author:** Shakir A, Ghoreifi A, 2020
Title: Feasibility, technique and perioperative outcomes of robotic parastomal hernia repair with biologic meh after cystectomy and ileal conduit diversion
Journal: Journal of urology; *conference abstract*: 2020 Annual Meeting of the American Urological Association. United States.
Patients: 7 patients undergoing robotic repair for parastomal hernia after ileal conduit urinary diversion
Intervention: robotic keyhole repair
Outcome: postoperative 90-day complications, intraoperative complications, operative time

E-Mail, no additional data available.
EXCLUSION: conference abstract; no additional data available.
(not matching inclusion criteria)

(34)**Author:** Sharma DM, Mishra V 2009
Title: Long term outcome of parastomal hernia repair in patients treated for benign urological disease.
Journal: BJU International; conference abstract: British Association of Urological Surgeons 2009 Annual Scientific Meeting. Glasgow United Kingdom
Patients: undergoing repair for parastomal hernia after benign indication for urinary diversion; *subgroup* 20 ileal conduit patients, 3 continent urostoma
Intervention: parastomal hernia repair, not specified
Outcome: rate of complication, recurrence

E-Mail, no response
EXCLUSION: conference abstract + subgroup; no specific/additional data available
(not matching inclusion criteria)

(35)**Author:** Tatay F, Anoro M, 2016

Title: New technique for parastomal hernia repair: "IVO technique"

Journal: Hispoanoamericana de Hernia; Spanish article

Patients: patients requiring parastomal hernia repair; *subgroup* of 9 ileal conduit patients

Outcome: feasibility?

E-mail not available.

EXCLUSION: subgroup, no specific data available.

(not matching inclusion criteria)

(36)**Author:** Tran H, Turingan I, 2014

Title: Single-port laparoscopic parastomal hernia repair with modified sugarbaker technique

Journal: Journal of the Society of Laparoendoscopic surgeons

Patients: patients requiring parastomal hernia repair, *subgroup* of 4 ileal conduit repairs

Intervention: modified sugarbaker repair

Outcome: recurrence, interoperative complications, postoperative complications, length of stay

EXCLUSION: insufficient patient numbers

(37)**Author:** Tully K, Von Bodman C 2017

Title: Evaluation of a novel three-dimensional funnel mesh for parastomal hernia repair after ileal conduit diversion

Journal: Journal of Urology; *conference abstract*: 112th Annual Meeting of the American Urological Association, AUA 2017. United States

Patients: 40 patients with clinically significant hernia after ileal conduit

Intervention: reduction of hernia sac, followed by mesh placement

Outcome: postoperative complications, recurrence

EXCLUSION: duplication (Tully 2019)

(38)**Author:** Tully KH, Roghmann F 2019

Title: Parastomal hernia repair with 3-D Mesh implants after radical cystectomy and ileal conduit urinary diversion – A single center experience using a purpose made alloplastic mesh implant

Journal: Urology

Patients: 40 patients with clinically significant hernia after ileal conduit

Intervention: reduction of hernia sac, followed by mesh placement

Outcome: postoperative complications, recurrence

INCLUSION: 40 patients undergoing repair

(39)**Author:** Velzaco C, Garvey E, 2016

Title: Laparoscopic parastomal hernia repair: a single center updated review

Journal: Hernia; *conference abstract*: 17th Annual Hernia Repair. Washington, DC United States

Patients: patients undergoing laparoscopic parastomal hernia repair at a single center; *subgroup* of 16 ileal conduit repairs

Intervention: sugarbaker/keyhole/relocation
Outcome: postoperative complications, intraoperative outcomes, recurrence

EXCLUSION: duplication (+ subgroup), same as No 6

(40)**Author:** Von Bodmann C, Brock M, 2012
Title: Parastomal hernia repair following ileal conduit urinary diversion using a novel funnel mesh implant
Journal: Journal of Urology; *conference abstract*: 2012 Annual Meeting of the American Urological Association, AUA. Atlanta, GA United States.
Patients: 13 patients with parastomal hernia after ileal conduit
Intervention: novel mesh implant specifically designed with a central penetration point and funnel for intestinal parts aiming to minimize penetration; intraperitoneal sublay
Outcome: recurrence, postoperative complications

E-Mail, no additional data available.
EXCLUSION: conference abstract; no additional data available
(not matching inclusion criteria)

(41) **Author:** Warren J, Grant A 2015
Title: Open retromuscular repair of parastomal hernias
Journal: Diseases of the colon and rectum; *conference abstract*: Annual Meeting of the American Society of Colon and Rectal Surgeons, ACSRS 2015. Boston
Patients: patients with symptomatic parastomal hernia; 11% (+-7) ileal conduit
Intervention: retromuscular repair
Outcome: surgical site infection, surgical site occurrence, hernia recurrence

E-Mail, too few additional data available.
EXCLUSION: conference abstract + subgroup;
(not matching inclusion criteria)

(42)**Author:** Ypsilantis E, George M, 2015
Title: Outcome of parastomal hernia repair with a modified sugarbaker technique
Journal: colorectal disease; *conference abstract*: 10th Scientific and Annual Meeting of the European Society of Coloproctology. Dublin Ireland.
Patients: patients requiring hernia repair at a tertiary center, *subgroup* of 15 ileal conduit patients
Intervention: modified sugarbaker technique
Outcome: postoperative morbidity, recurrence

E-Mail, no response
EXCLUSION: conference abstract + subgroup; no additional/specific data available
(not matching inclusion criteria)

(43)41st Annual Congress of the European Hernia Society: Evidence, Innovation, Education; September 11–14, 2019—Hamburg, Germany:
Author: Martin, J. A.
Title: Parostomal hernias: a more and more surgical challenge

Journal: presented at the 41st Congress of the European Hernia Society
Patients: 60 patients who underwent surgery for parastomal hernia, *subgroup* of 12 ileal conduit patients
Intervention: eventroplasty with Dynamesh IPST implant, 3D-preshaped mesh
Outcome: recurrence, safety

E-mail, no additional data available.
EXCLUSION: conference abstract + subgroup; no additional/specific data available.
(not matching inclusion criteria)

(44)41st Annual Congress of the European Hernia Society: Evidence, Innovation, Education; September 11–14, 2019—Hamburg, Germany:
Author: Borshchigov, M., Gröger, C.
Title: Laparoscopic treatment of parastomal hernias: a single center experience with the Pauli procedure
Patients: patients requiring surgery for parastomal hernia, *subgroup* of 6 patients with urostomy
Intervention: laparoscopic Pauli procedure in 5 patients
Outcome: postoperative hospital stay and operative time, complications

E-Mail, no additional/specific data available
EXCLUSION: conference abstract + subgroup; no additional/specific data available.
(not matching inclusion criteria)

(45) American Hernia Society Annual Meeting 2020:
Author: Mäkäräinen-Uhlbäck, E., Vironen, J.
Title: Keyhole versus Sugarbaker techniques in parastomal hernia repair at bricker conduit: a retrospective nationwide cohort study with long-term follow-up
Patients: patients requiring primary repair of a ileal conduit parastomal hernia, 34 patients
Intervention: Keyhole (n=25), Sugarbaker (n=6)
Outcome: primary outcome was parastomal hernia recurrence, secondary outcomes were complication and reporation rate

E-Mail, full article will be published in near future
EXCLUSION: conference abstract, no additional data available
(not matching inclusion criteria)
